# Prediction of functional characteristics of gari (cassava flakes) using near-infrared reflectance spectrometry

**DOI:** 10.3389/fchem.2023.1156718

**Published:** 2023-05-10

**Authors:** Michael Adesokan, Emmanuel Oladeji Alamu, Segun Fawole, Busie Maziya-Dixon

**Affiliations:** ^1^ Food and Nutrition Sciences Laboratory, International Institute of Tropical Agriculture (IITA), Ibadan, Nigeria; ^2^ International Institute of Tropical Agriculture, Southern Africa Research and Administration Hub (SARAH) Campus, Lusaka, Zambia

**Keywords:** cassava, gari, functional properties, NIRS, prediction model

## Abstract

Gari is a creamy, granular flour obtained from roasting fermented cassava mash. Its preparation involves several unit operations, including fermentation, which is essential in gari production. Fermentation brings about specific biochemical changes in cassava starch due to the actions of lactic acid bacteria. Consequently, it gives rise to organic acids and a significant reduction in the pH. Consumer preferences for gari are influenced by these changes and impact specific functional characteristics, which are often linked to cassava genotypes. Measurement of these functional characteristics is time-consuming and expensive. Therefore, this study aimed to develop high-throughput and less expensive prediction models for water absorption capacity, swelling power, bulk density, and dispersibility using Near-Infrared Reflectance Spectroscopy (NIRS). Gari was produced from 63 cassava genotypes using the standard method developed in the RTB foods project. The prediction model was developed by dividing the gari samples into two sets of 48 samples for calibration and 15 samples as the validation set. The gari samples were transferred into a ring cell cup and scanned on the NIRS machine within the Vis-NIR range of 400–2,498 nm wavelength, though only the NIR range of 800–2,400 nm was used to build the model. Calibration models were developed using partial least regression algorithms after spectra pre-processing. Also, the gari samples were analysed in the laboratory for their functional properties to generate reference data. Results showed an excellent coefficient of determination in calibrations (R^2^_Cal_) of **0.99**, **0.97**, **0.97**, and **0.89** for bulk density, swelling power, dispersibility, and water absorption capacity, respectively. Also, the performances of the prediction models were tested using an independent set of 15 gari samples. A good prediction coefficient (R^2^ pred) and low standard error of prediction (SEP) was obtained as follows: Bulk density (**0.98**), Swelling power (**0.93**), WAC (**0.68**), Dispersibility (**0.65**), and solubility index (**0.62**), respectively. Therefore, NIRS prediction models in this study could provide a rapid screening tool for cassava breeding programs and food scientists to determine the food quality of cassava granular products (Gari).

## 1 Introduction

Cassava (*Manihot esculenta* Crantz) is an essential staple crop grown throughout the tropics by more than 800 million people (Teeken et al., 2018), and it is the third primary source of calories after rice and maize (Adetoro et al., 2018). Nigeria, Brazil, Thailand, Indonesia, and the Democratic Republic of Congo (DRC) are responsible for about 60% of cassava production, and Nigeria is the leading producer (Ohimain, 2015). Cassava’s global production in 2015 and 2016 was estimated to be around 281 million tons and 288.4 million tons, respectively (FAO, 2016), while Nigeria was reported to have engaged over four million farmers in cassava production (FAO, 2018). Cassava roots are drought tolerant; hence they are widely cultivated for their ability to withstand harsh environmental and agronomic conditions. As a result of its ability to survive in the face of adverse climatic conditions, cassava is often called Africa’s food insurance (Jarvis et al., 2012; Belalcazar et al., 2016). The root crop is the second most important food staple in sub-Saharan Africa, while in Nigeria, it is a primary staple food which is consumed by more than 100 million people daily because it is an efficient and easy source of carbohydrate food energy (Tarawali et al., 2012; Adetoro et al., 2018). Due to the versatility of the crop, it can be prepared into various foods, used as animal feeds, and produce as industrial materials such as starch (Bechoff et al., 2018). It has been used to manufacture plywood, paper, textiles, and adhesives (Tonukari et al., 2015). In the food industry, cassava has been processed into numerous products like bread, pasta, and couscous-like products (Mtunguja et al., 2019). In Nigeria, the major cassava-based products are gari, fufu and lafun, produced and consumed by the farmers (Teeken et al., 2018).

Gari, one of the significant products from cassava roots in the West Africa sub-region, is a dry, crispy, and granular food product (Udoro et al., 2014; Awoyale et al., 2021a). It is the most traded cassava food product in West and Central Africa, with Nigeria as the largest producer (FAO, 2018). The cassava roots are peeled, washed, and grated during gari production. The grated mash is then dewatered by pressing, fermented (optional), sieved and roasted (Escobar et al., 2018). The optional fermentation and addition of palm oil influence the classification of gari, usually “Ijebu gari” and “yellow gari.” Ijebu gari is produced by fermenting the cassava mash before roasting, while yellow gari is processed without fermentation but by adding red palm oil before roasting (Erukainure et al., 2022). The granular product is a versatile and convenient food due to its cheapness, ease of storage, long shelf-life, and short preparation time for consumption, making it extremely popular among urban dwellers in Nigeria and other West African countries (Irtwange and Achimba, 2009). It is the most consumed cassava food product in West Africa, and Nigeria is the largest consumer (Ndjouenkeu et al., 2021). In Nigeria, gari production has contributed immensely to the nation’s economic growth, a substantial portion of small and medium enterprises (SMEs) is occupied by gari processing firms (Ogundipe et al., 2013). The versatility of gari is reflected in the various ways it can be consumed, such as soaking in cold water and consumed directly with sweeteners, groundnut and fish. It can be consumed as a dough by sprinkling it into a measured quantity of boiling water with continuous stirring until a consistent dough is formed. The dough, popularly called “eba,” is the most widely eaten form of gari in Nigeria (Irtwange and Achimba, 2009; Adinsi et al., 2019; Teeken et al., 2021). These primary forms in which gari is consumed take advantage of one of its functional properties, specifically the swelling power, which is a critical factor that influences the overall acceptability of the product by consumers (Ndjouenkeu et al., 2021, Becerra Lopez-Lavalle et al., 2018). The functional properties of food materials, such as bulk density, water absorption capacity, swelling power, and dispersibility, often indicate how the food materials interact with other food components, affecting food quality and consumer acceptability (Awoyale et al., 2021b). The bulk density of food material is a crucial determinant of the packaging materials suitable for such food material and influences its handling. Whereas the extent to which gari swells affects its final quality (Awoyale et al., 2020). Also, the swelling power of starch indicates its specific functional properties when utilized in food products, which is often a function of the amylopectin content of the starch (Noranizan et al., 2010). The functional properties of cassava food products are essential to the breeders because it influences their acceptability by processors and consumers; hence the need for a technology that can rapidly evaluate these properties.

One of the significant obstacles to developing rapid screening and quality control in the agricultural and food industry is the need for more simple, reliable, and non-destructive methods for determining chemical composition in agricultural products (Cozzolino et al., 2013). Near-infrared reflectance spectroscopy (NIRS) is a non-destructive, high throughput technique which measures the interactions between electromagnetic radiation and vibrational properties of chemical bonds (Alamu et al., 2021). It is an important method that has led to more efficient breeding as it offers the advantage of characterizing a more significant number of samples in shorter time than other wet laboratory techniques (Belalcazar et al., 2016). NIRS spectroscopy determined whole-grain barley’s swelling properties and water solubility (Cozzolino et al., 2013). Mbanjo et al. (2021) also reported that NIRS technology could predict cassava or cassava-based products’ functional and physicochemical properties. Other applications of NIRS were reported in literature, Chen et al. (2014) have developed a stable quantitative model for the rapid quality evaluation of *Lonicera japonica* based on its ethanol precipitation process. The protein and glucose content of flour from roots and tubers were determined using NIRS (Masithoh et al., 2021). Also, short wavelength near infrared reflectance spectroscopy was used to determine the starch content of fresh cassava roots (Bantadian et al., 2020).

Apart from its contributions in the agricultural and food industries, NIRS has also found many practical applications in other industries such as medicine, forensic science, and pharmaceuticals (Heise, 2021; Sacré et al., 2021; Chen et al., 2022). Several authors have reported the application of NIRS for predicting the quality parameters of cassava and its products (Sanchez et al., 2014; Fu et al., 2017; Ikeogu et al., 2017; 2019; Su and Sun, 2017; Alamu et al., 2019). However, no work has been reported on using NIRS to predict gari’s functional properties. Therefore, this study seeks to evaluate the application of NIRS in predicting the selected functional properties of gari.

## 2 Materials and methods

### 2.1 Source of materials and sample preparation

**FIGURE 1:**
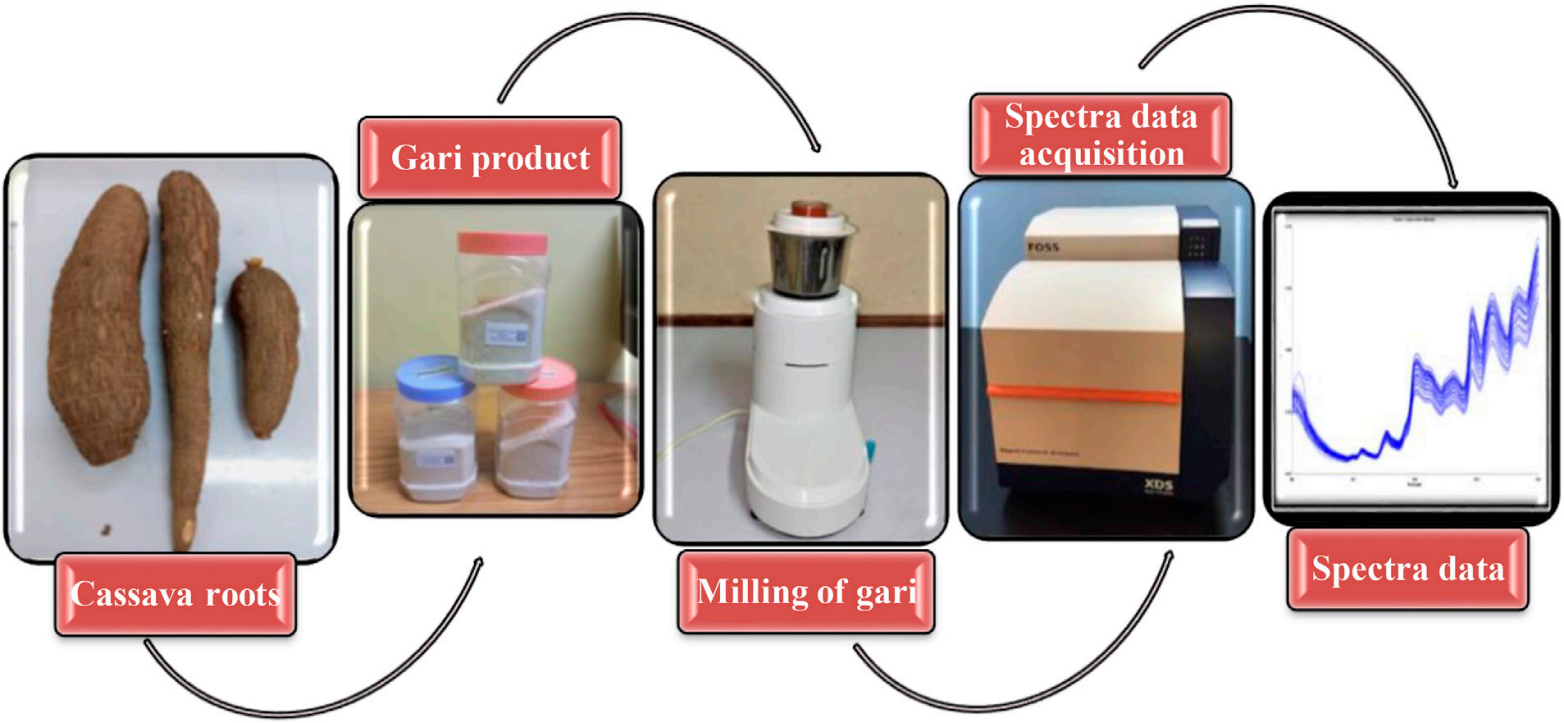
A workflow of sample processing and analysis using NIRS.

The cassava roots (which were processed into gari) were obtained from the experimental field plots of the International Institute of Tropical Agriculture (IITA). Sixty-three gari samples were used for this study, and to prepare the gari (cassava flakes), the fresh cassava roots were peeled, washed, and grated using a mechanical grater. The grated mash was transferred into a jute bag and pressed under a jack for 72 h to eliminate the water. Pressed mash was collected, sieved, and roasted under a controlled heat source until desired gari quality was formed. Roasted gari was allowed to cool and then milled using an electric laboratory blender. The finely ground gari was packed in well-labelled plastic containers and transferred for subsequent analysis. See Figure 1.

### 2.2 Laboratory analysis

Water absorption capacity (WAC; g mL^−1^) and Dispersibility (%) of gari were determined using methods described by AOAC (2006). For WAC, about 1 g of each gari was transferred to a clean 15 mL centrifuge tube with 10 mL of distilled water, centrifuged at 512 g for 15 min (GLC-1, Chicago, United States). After centrifugation, the supernatant was decanted, and the weight of the sediment was taken to determine the WAC. Dispersibility was determined by weighing 10 g of the sample into a 100-mL measuring cylinder and distilled water added to reach a volume of 50 mL. The mixture was stirred vigorously, particles were allowed to settle for 3 h, and the percentage volume of settled particles was calculated to determine the percentage dispersibility. Bulk density (BD, g mL^−1^) was determined using the method that Ashraf et al. (2012) reported, where 10 g of the sample was weighed into a 50 mL graduated measuring cylinder and tapped gently on a benchtop about ten times. Bulk density was recorded as grams per millilitre. The swelling power (SP; g mL^−1^) and solubility index (SI, %) were determined using the method described by Riley et al. (2006) with a slight modification, where 50 mL of distilled water was added to 1 g of the sample in a centrifuge tube and incubated for 30 min in a water bath at 95°C. The mixture was centrifuged at 512 g for 15 min, and the difference in the mass of the sediment calculated the mass of soluble substances in the supernatants.

### 2.3 Spectra collection and calibration development

**FIGURE 2:**
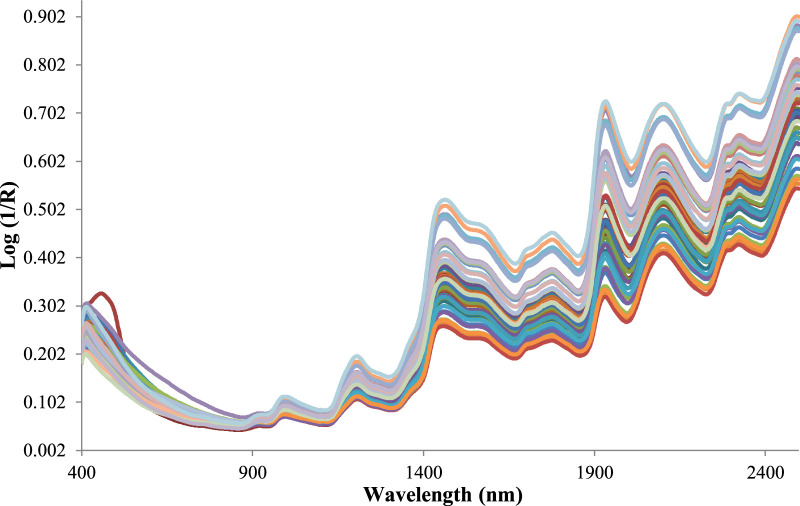
Near infrared reflectance spectrum for gari samples**. (**NIR wavelength region of 800–2,400 nm was used for the model).

The gari samples were scanned in duplicate within the wavelength range of 400–2,498 nm, registering the absorbance values log (I/R) at 0.5 nm intervals for each sample and using a NIRS monochromator (model FOSS XDS, solid module) and a stationary cell cup. Though only the NIR range of 800–2,400 nm was used to build the model. Data and statistical analyses were performed using Win-ISI 4.9 software (Infrasoft International and FOSS, Hillerod, Denmark). NIR spectra are often affected by instrumental noise, sample particle size, and other environmental factors; therefore, preprocessing of the spectra is important before model development. Using appropriate preprocessing methods is critical to eliminating interferences and background noise, which will help to improve the model prediction accuracies. In this experiment, the spectra data were subjected to various preprocessing methods to correct the effects of light scattering and increase the signal-to-noise ratio. Several mathematical treatments, including 1,4,4,1; 2,10,10,2; 2,10,5,1; 2,5,5,1; 1,25,10,1; 1,10,10,1; and 0,0,1,1 respectively and combined with standard normal variate and detrend (SNVD) was implemented to optimise the equation. Model performance from each pretreatment was evaluated to decide the best treatment that gives a reliable model. The first and second numbers represent the derivative and gap, while the last two are smoothings. Outliers’ eliminations were conducted using the neighborhood Mahalabonis distance (NH) and the GH, which is the distance of each spectrum from the mean spectrum of the sample populations. The NH calculates how close each sample is to every other sample in the population. The GH determines whether the calibration model can accurately forecast the value of an unknown sample and allow for the removal of unnecessary spectra from the calibration population. Outliers are eliminated based on the standard residuals with a cutoff of GH > 2.5 and NH < 0.6. The calibration was set up using the first derivative of SNVD corrected spectra, calculated on four data points, and smoothed using Savitzky–Golay polynomial smoothing on the four data points. The calibration model was developed using the modified partial least square (MPLS) regression algorithm using a spectral range between 800 and 2,400 nm (Figure 2). A set of 63 samples with their reference results were split into 48 calibrations and 15 validation sets. The spectra data for the samples collected on the NIRS device correlated with the reference values for each constituent. The model developed was tested using an independent set of samples to compare the prediction of the functional properties and the results of standard laboratory methods.

### 2.4 Model performance evaluations

The performance of the calibration model was evaluated using performance indicators such as the coefficients of determination in calibration (R^2^cal), coefficients of determination in prediction (R^2^pre), root mean square error in calibration (RMSEC), root mean square error in prediction (RMSEP), and cross-validation (RMSECV) and residual prediction deviation (RPD). The model’s performance is better when R^2^ is close to 1 and RMSE is close to zero. Higher RPD also indicates a good prediction model. According to He et al. (2018), R^2^ values between 0.61 and 0.80 indicate a decent model for prediction, whereas R^2^ values above 0.90 show an outstanding model for more accurate determination. Smaller RMSEs indicate better model fitting. RPD values between 1.8 and 2.0 is good for quantitative predictions; whereas RPD between 2.0 and 2.5 implies very good and, RPD >2.5 indicates an excellent prediction (Chadalavada et al., 2022).

### 2.5 Statistical analysis and software package

All spectra preprocessing, calibration model development and prediction analysis were conducted using the WIN ISI Software Version 4.0.

## 3 Results and discussion

The summary statistics of the functional properties of the analyzed gari samples are shown in Table 1. Swelling power and Solubility index ranged from 6.05% to 16.70% and 3.33% to 16.94%, respectively, with average values of 13.63% and 9.16%. The average swelling power (13.63%) reported in this study is comparable with values reported by Awoyale et al., 2021b. Swelling power is the ability of gari particles to absorb water and swell; a quality gari should swell considerably well (Udoro et al., 2014). Awoyale et al. (2020) also reported that a quality gari should swell three times its original volume. Gari is often consumed by soaking in water. Therefore, the swelling of gari is a critical quality criterium consumer’s desire. Also, the results showed a range of 29%–72% and 0.70%–66.67% for dispersibility and bulk density, respectively. The functional properties of food are essential to the end users because they affect food behaviours during preparation and storage (Awoyale et al., 2020). Some functional properties of food also depend predominantly on the product’s particle size and other physical characteristics (Nwancho et al., 2014). The bulk density of food material determines its handling requirement and packaging materials. The particle size of the food materials influences their bulk density because they are inversely related (Nwancho et al., 2014). Lower BD reported in this study implies that more gari could be packaged in a specific container volume, decreasing the space occupied during packaging (Komolafe and Arawande, 2010). Average dispersibility was in line with values reported by Awoyale et al. (2020) while the average WAC in this study was higher than the value reported by Udoro et al. (2014).

**TABLE 1:** Summary statistics of functional properties of gari samples.

N = 63				
Constituent	Minimum	Maximum	Mean	SD
SP	6.05	16.70	13.63	2.53
SI	3.33	16.94	9.16	3.75
DISP	29.00	72.00	51.74	14.00
BD	0.70	66.67	23.06	30.09
WAC	375.95	658.83	511.09	68.32

N, sample number; SD, standard deviation.

**FIGURE 3:**
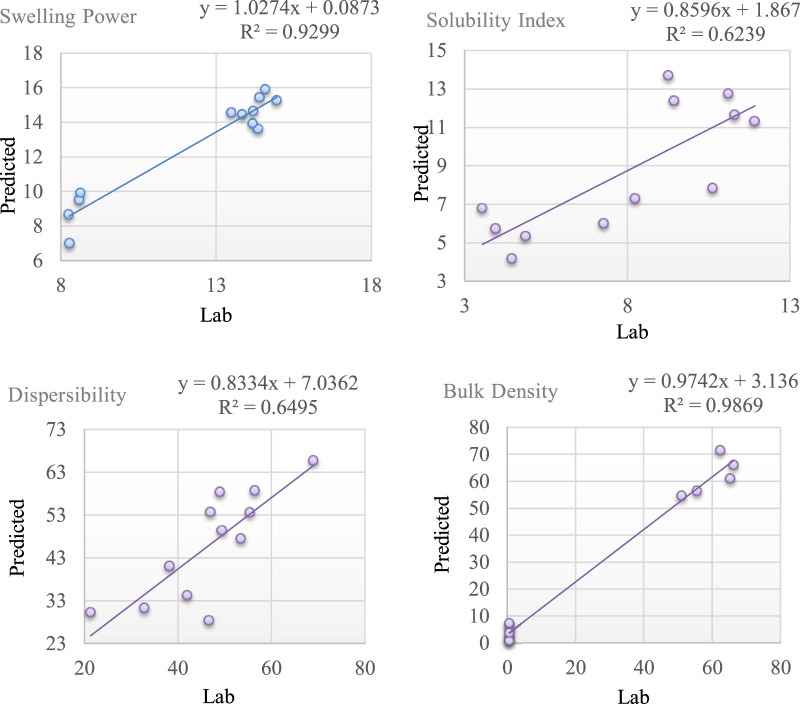
Graph of predicted vs. laboratory values for the functional properties of gari samples.

Tables 2, 3 show the calibration and validation statistics of the functional properties of gari. The calibration of gari samples using 48 samples with wide variations in their functional properties shows that NIRS closely correlates with the standard laboratory analysis method. Spectra pretreatments are an important step in model development to eliminate other factors, such as instrumental noise; and detector drift which could interfere with model performance. Therefore, this study tested several pretreatments to establish the best equation. Table 4 shows different treatments and their respective performance statistics, including the model with no treatments (0,0,1,1). The coefficient of determination in calibration (R^2^cal) of SP for all the mathematical treatments tested (0,0,1,1; 2,10,5,1; 1,4,4,1; 1,25,10,1; 1,10,10,1 and 2,5,5,1) were 0.95, 0.98,0.97,0.87,0.95 and 0.97 respectively. The best R^2^cal for SP was obtained from 2,10,5,1 pre-treatment followed by 1,4,4,1 and 2,5,5,1, but the standard error of cross-validation in other treatments was higher than 2,5,5,1. Therefore, 2,5,51 was preferred as the appropriate treatment for Swelling power. Comparatively, the pre-treatment 2,5,5,1 gives better prediction performance regarding high R^2^cal and low SECV for most functional properties. Bulk density had the highest R^2^cal of 0.99, followed by SP (0.97) and Dispersibility (0.97) for treatments 2,5,51. The least R^2^cal was obtained for the solubility index across all the treatments tested. Lu et al. (2006) reported R^2^cal of 0.92 and 0.88 for Swelling power and solubility of sweet potato starch using NIR spectrometry; the results for SP (0.97) and SI (0.88) for gari reported in this study are similar to their findings. The coefficient of determination in prediction should typically be in the range of 0.66–0.81 for the NIR prediction to be adequate for quick screening. It should have a range of 0.83–0.90 for quality control and an accurate determination (Lebot et al., 2013). Therefore, the R^2^cal for the parameters analyzed in this study is considered adequate and suitable for screening large samples in breeding programs. The model’s performance was further tested using an independent set of samples not included in the calibration sets by comparing the results from the standard laboratory method with the predicted values using the developed model. The coefficient of determination in validation (R^2^pred) followed the same trend as the calibration statistics; the BD had the highest R^2^pred of 0.98, followed by SP (0.93). In contrast, SI had the lowest R^2^pred of 0.62 (Figure 3).

**TABLE 2:** Calibration statistics for the functional properties of gari samples.

	Calibration	N = 48		
Constituent	SEC	R^2^cal	SECV	Outliers
SP	0.43	0.97	1.12	0
SI	2.29	0.64	2.46	1
DISP	2.31	0.97	6.59	0
BD	3.36	0.99	6.38	0
WAC	21.10	0.89	43.34	5

N, sample number; SEC, standard error of calibrations; SECV, Standard error of cross-validation; R^2^cal, coefficient of determination in calibrations.

**TABLE 3:** Validation statistics for the functional properties of gari samples.

Validation N = 15						
Constituent	R^2^pred	SEP	Bias	Slope	Outliers	RPD
SP	0.93	0.89	0.67	0.91	3	2.6
SI	0.62	2.16	1.47	0.73	3	2.4
DISP	0.65	7.50	3.96	0.78	3	2.1
BD	0.98	4.42	3.83	0.98	3	2.2
WAC	0.68	40.89	26.00	26.48	3	1.9

N, sample number; SEP, standard error of prediction; R^2^pred, coefficient of determination in validations. RPD, Ratio of prediction to standard deviation of reference values.

**TABLE 4:** Model optimization using different spectra pre-treatments.

	0,0,1,1			2,10,5,1			1,4,4,1		
Constituent	SEC	R^2^cal	SECV	SEC	R^2^cal	SECV	SEC	R^2^cal	SECV
SP	0.59	0.95	1.08	0.38	0.98	0.83	0.46	0.97	1.03
SI	2.36	0.56	2.45	2.28	0.59	2.37	2.29	0.59	2.37
DISP	5.42	0.85	6.97	4.47	0.90	6.21	4.47	0.90	5.71
BD	3.12	0.99	5.22	1.88	0.99	4.90	1.90	0.99	4.99
WAC	43.79	0.58	47.79	46.33	0.53	48.07	45.68	0.55	47.40

	1,25,10,1			1,10,10,1			2,5,5,1		
	SEC	R^2^cal	SECV	SEC	R^2^cal	SECV	SEC	R^2^cal	SECV
SP	0.94	0.87	1.16	0.59	0.95	1.08	0.17	0.97	0.81
SI	2.29	0.59	2.37	2.36	0.56	2.45	2.27	0.64	2.38
DISP	4.48	0.90	5.58	5.42	0.85	6.97	5.03	0.97	6.45
BD	2.74	0.99	5.51	3.12	0.99	5.22	1.60	0.99	4.99
WAC	45.46	0.55	47.05	43.79	0.58	47.79	46.05	0.89	48.07

SEC, standard error of calibrations; SECV, Standard error of cross-validation; R^2^cal, coefficient of determination in calibrations.

The performance of a model is not determined only by the coefficient of determination but also by the closer the standard prediction error is to zero. The least SEP was reported for SP, followed by SI and BD. The R^2^cal of calibration models could be affected by several factors, such as lack of genetic variability, poor representativeness of the samples within the calibration data sets and the accuracy of the reference method (Alamu et al., 2022). Therefore, the relatively low R^2^cal observed for SI could be improved by increasing the training population used for the calibration. Also, the low SEC (2.29) and SEP (2.16) values for SI indicate the potential to improve the model by introducing more samples into the calibration data set. Model performance was also evaluated by the bias in the prediction statistics; bias indicated similarities between the reference results and the predicted values. The ideal value for bias should be zero, that is, when the reference results of a parameter are the same as the predicted values. The bias becomes negative when the model underestimates the constituent’s information, while it is positive when the values are overestimated. The functional properties in this study were not underestimated, but SP, SI and Dispersibility were slightly overestimated by the positive values of the bias. Though the RDP, which is the ratio of the standard deviation of the reference value and the standard error of prediction, is greater than 2 for all the parameters except for WAC, showing that the model is promising in the accurate prediction of most of the parameters.

## 4 Conclusion

NIRS offers a high throughput and less expensive alternative to the elaborate and time-consuming wet chemical analysis methods in the laboratory for determining the functional composition of gari. These functional parameters are critical indicators of the final product quality of gari, which influences consumers buying decision. A rapid method for their determination is important for breeding programs and processors to assess the quality of the products especially when larger number is to be considered. This study has shown that Near-infrared reflectance spectroscopy has the potential to predict the quality parameters of gari by using a few samples sets but with wide variation in their functional properties. The model developed with R^2^cal above 0.90 can be applied by breeders and food scientists for rapid screening of the functional properties of gari, especially swelling power and bulk density.

## Data Availability

The raw data supporting the conclusion of this article will be made available by the authors, without undue reservation.
